# X-Nuclei NMR Self-Diffusion Studies in Mesoporous Silica Foam and Microporous MOF CuBTC

**DOI:** 10.3390/ma5040617

**Published:** 2012-04-12

**Authors:** Stefan Schlayer, Anne-Kristin Pusch, Friederike Pielenz, Steffen Beckert, Mikuláš Peksa, Carsten Horch, Lutz Moschkowitz, Wolf-Dietrich Einicke, Frank Stallmach

**Affiliations:** 1 Institute of Experimental Physics I, Universität Leipzig, Linnéstr. 5, Leipzig 04103, Germany; E-Mails: s.schlayer@physik.uni-leipzig.de (S.S.); akpusch@physik.uni-leipzig.de (A.-K.P.); fpielenz@yahoo.de (F.P.); s.beckert@physik.uni-leipzig.de (S.B.); mikulas.peksa@uni-leipzig.de (M.P.); horch@physik.uni-leipzig.de (C.H.); lumo@physik.uni-leipzig.de (L.M.); 2 Institute of Technical Chemistry, Universität Leipzig, Linnéstr. 4, Leipzig 04103, Germany; E-Mail: einicke@chemie.uni-leipzig.de

**Keywords:** probe design, diffusion, ^7^Li, ^13^C, ^133^Cs, PFG NMR, CuBTC, MCF

## Abstract

A standard X-observe NMR probe was equipped with a *z*-gradient coil to enable high-sensitivity pulsed field gradient NMR diffusion studies of ${\mathrm{Li}}^{+}$ and ${\mathrm{Cs}}^{+}$ cations of aqueous salt solutions in a high-porosity mesocellular silica foam (MCF) and of ${\mathrm{CO}}_{2}$ adsorbed in metal-organic frameworks (MOF). The coil design and the necessary probe modifications, which yield pulsed field gradients of up to $\pm 16.2\;{\mathrm{Tm}}^{-1}$, are introduced. The system was calibrated at ${}^{2}H$ resonance frequency and successfully applied for diffusion studies at ${}^{7}\mathrm{Li}$, ${}^{23}\mathrm{Na}$, ${}^{13}C$ and ${}^{133}\mathrm{Cs}$ frequencies. Significant reductions of the diffusivities of the cations in ${\mathrm{LiCl}}_{ac}$ and ${\mathrm{CsCl}}_{ac}$ solution introduced into MCFs are observed. By comparison of the diffusion behavior with the bulk solutions, a tortuosity of the silica foam of 4.5±0.6 was derived. Single component self-diffusion of ${\mathrm{CO}}_{2}$ and ${\mathrm{CH}}_{4}$ (measured by ${}^{1}H$ NMR) as well as self-diffusion of the individual components in ${\mathrm{CO}}_{2}$/${\mathrm{CH}}_{4}$ mixtures was studied in the MOF CuBTC. The experimental results confirm high mobilities of the adsorbed gases and trends for diffusion separation factors predicted by MD simulations.

## 1. Introduction

Diffusion processes of liquids and gases under the influence of internal surfaces of porous materials are of fundamental importance for understanding host-guest-interaction and of practical relevance for the prediction of material properties. Under thermodynamically equivalent conditions with respect to temperature and pressure, the interactions with the internal surface of the porous host material generally reduce the mobility of the guest molecules (the pore fluid) compared to the free (bulk) fluid. Therefore, and due to the restricted possibilities to observe the pore fluids, diffusion processes in the pore space are more difficult to study than in the bulk fluid phase.

To measure diffusion in porous systems, many experimental approaches such as modern IR microscopy [1,2] require a sudden change of external conditions in the fluid phase (concentration, pressure) [3]. By observing and analyzing the response of the system in space and time, these methods yield non-equilibrium (transport) diffusion data. Quasi elastic neutron scattering (QENS) [4,5] and pulsed field gradient nuclear magnetic resonance (PFG NMR) [6,7,8] are also suitable to observe molecular motion in the interior of the porous host system. These techniques observe nondestructively the self-diffusion under equilibrium conditions using beds or large monoliths of the porous host system.

Most of the PFG NMR diffusion studies in porous materials are performed with adsorbate molecules containing ${}^{1}$H hydrogen in the molecular structure. Compared to other (X) nuclei, the gyromagnetic ratio and signal intensity of the ${}^{1}H$ (proton) nucleus offer the best measurement conditions [9]. X-nuclei provide better chemical shift contrast than the ${}^{1}$H nucleus but offer only a reduced sensitivity for diffusion coefficients and mean square displacements since this diffusion sensitivity is proportional to the square of the gyromagnetic ratio of the observed nucleus [10]. In modern application of porous materials such as, e.g., for gas separation [11,12,13,14], ion conduction [15] or environmental remediations [16,17], the diffusing objects of interest may not contain ${}^{1}$H nuclei. Thus, it is a challenging task for experimental NMR diffusion research to improve the diffusion sensitivity also for X-nuclei.

In this paper, we introduce a modification of a Bruker X-observe NMR probe with a Maxwell-pair *z*-gradient coil to enable high-sensitivity pulsed field gradient NMR self-diffusion studies with a wide range of X-nuclei. ${}^{7}$Li, ${}^{13}$C and ${}^{133}$Cs PFG NMR experiments are performed in different porous host systems. The self-diffusion of Li${}^{+}$ and Cs${}^{+}$ cations in aqueous solutions of LiCl and CsCl introduced into a high-porosity mesocellular silica foam (MCF) [18,19] are measured and compared to the bulk diffusivities revealing the restricting influence of the solid silica host matrix on the diffusion pathway of the cations. These studies serve both the experimental test of the developed probe with a porous material and the characterization of the internal diffusion resistance of the silica matrix of the MCF. Additionally, the self-diffusion of carbon dioxide and methane is investigated in the microporous MOF CuBTC [20] using ${}^{13}$C NMR and ${}^{1}$H NMR, respectively. The data are compared to MD simulations of diffusion and of diffusion separation factors [21,22] and provide experimental verifications of the results of the computer simulations.

## 2. Results and Discussion

### 2.1. Performance of the Modified X-Observe Probe for Diffusion Studies

A Maxwell-pair type gradient coil [8,10] was designed to fit a commercial X-observe NMR probe (type WB 400, Bruker Germany). The coil was manufactured and mounted on the probe. The necessary gradient current leads were added, including RF blocking capacitors and air cooling to dissipate thermal energy generated during gradient operation. The gradient coil was designed in such a way that (1) it accommodates well the standard X-nuclei RF coil of the probe including its thermal insulation from the gradient coil support, (2) it produces a high current-to-gradient conversion factor and (3) the gradient is homogeneous over the anticipated cylindrical sample volume of 1cm in diameter and in height. Additionally, magnetic fields generated by the coil on the positions of the outer metal housing of the probe should be as small as possible to prevent disturbing influences of eddy currents during the rising and falling slopes of the field gradient pulses.

**Figure 1:**
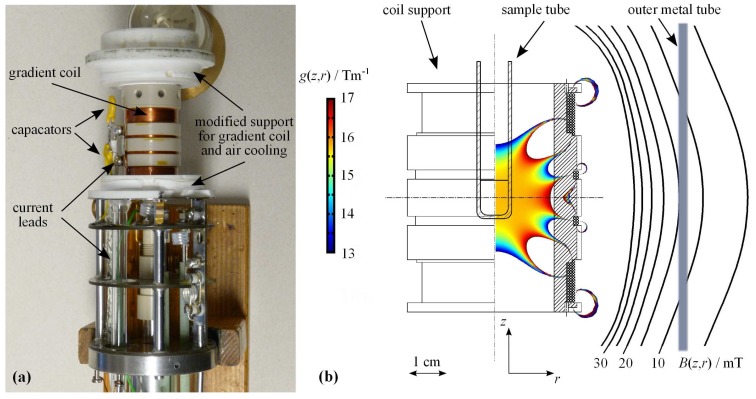
X-observe NMR probe equipped with a *z*-gradient coil. (**a**) Photograph of the top part of the NMR probe with its metal cover removed. Added and modified components are indicated; (**b**) Drawing of the gradient coil support with the color plot of the calculated field gradient g(z,r) at the NMR sample position and the contour plot of the stray field B(z,r) of the gradient coil at the position of the metal tube (outer probe cover). The spatial dependence of the gradient and field values are plotted for the maximum available gradient current of 100A.

Figure 1a displays a photograph of the gradient coil mounted on the top part of the probe. The RF coil and its thermal insulation are covered by the gradient coil and are not visible. Figure 1b shows the drawing of the designed gradient coil support. In the inner and outer parts, the drawing contains the color coded two-dimensional plot of the gradient intensity in *z*-direction g(z,r) and the contour plot of the stray field B(z,r), respectively. Due to the cylindrical symmetry of the gradient coil, the field gradient and the stray field are plotted only on half space in the zr-plane. Figure 1b provides a visual impression on the calculated intensities of g(z,r) and B(z,r) at a gradient current of 100A, which corresponds to the maximum available pulsed gradient current value on our NMR systems. Details of the calculation procedure for the gradient and the stray field and the coil manufacture are given in the Experimental Section.

The analysis of the variability of the *z*-gradient intensity g(z,r) showed that the calculated, spatially averaged current-to-gradient conversion factor *c* (g(t)=c·I(t), *I* gradient current) of the gradient coil is $\left(0.159\pm 0.004\right)\;T{\left(m\;A\right)}^{-1}$ over the anticipated sample volume. The strongest deviations from the averaged value to the higher band are observed at r≥4.5mm, which corresponds to the inner diameter of 10mm o.d. NMR sample tubes and, hence, does not contribute to the sensitive sample volume anymore. Thus, in agreement with the visual impression from Figure 1b, the field gradient is expected to have sufficient homogeneity over the sample volume for NMR self-diffusion studies.

The actually achieved current-to-gradient conversion factor and the coil performance with respect to gradient linearity and eddy current depends of the individual coil manufacture. Therefore, the coil was calibrated by measuring the spin echo attenuation of liquid deuterated water (${}^{2}H_{2}O$) using ${}^{2}H$ PFG NMR. For self-diffusion in liquid water, the spin echo NMR signal M(b) is expected to follow the dependence

(1) $$M\left(b\right)=M_{0}\exp\left(-bD\right)$$

where $M_{0}$ denotes NMR spin echo amplitude observed without pulsed field gradients and *D* is the self-diffusion coefficient of the molecules carrying the observed nuclei. The *b*-value depends on the applied pulse sequence, on the pulsed field gradient pattern g(t) and on the gyro-magnetic ratio *γ* of the observed NMR-active nucleus. It is determined via the so-called double integral (see e.g., [6,8,10]). For two rectangularly shaped pulsed field gradients of duration *δ*, intensity *g* and separation *Δ* applied in a primary or stimulated spin echo NMR sequences, one obtains

(2) $$b=\gamma^{2}{\left(g\delta \right)}^{2}\left(\Delta -\frac{1}{3}\delta \right)$$

**Figure 2:**
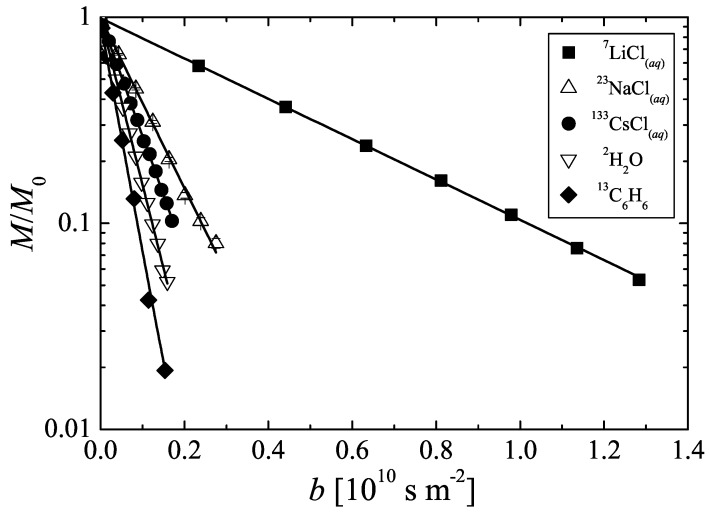
Pulsed field gradient calibration and performance tests of the X-observe probe equipped with a *z*-gradient coil for NMR diffusion studies. NMR spin echo attenuations due to self-diffusion of ${\mathrm{Li}}^{+}$, ${\mathrm{Na}}^{+}$, ${\mathrm{Cs}}^{+}$ in aqueous solutions as well as of bulk deuterated water and ${}^{13}C$ enriched benzene (see legend) observed using ${}^{7}\mathrm{Li}$, ${}^{23}\mathrm{Na}$, ${}^{133}\mathrm{Cs}$, ${}^{2}D$, ${}^{13}C$ and ${}^{2}H$ resonance, respectively.

The experimental ${}^{2}H$ PFG NMR spin echo attenuation curve ($M\left(b\right)/M_{0}$ over *b*) of deuterated water is plotted in Figure 2. In this semi-logarithmic presentation, the observed decay is linear, which means that the designed coil and the modified X-observe NMR probe follow the behavior expected from Equation 1. Weingärtner *et al.* [23] reported that ${}^{2}H_{2}O$ has a self-diffusion coefficient of $1.87\times 10^{-9}\;m^{2}s^{-1}$ at 298K. This value was applied to calibrate the current-to-gradient conversion factor *c* of the new probe in such a way that the slope of the ${}^{2}H$ PFG NMR spin-echo attenuation curve in Figure 2 corresponds to this known self-diffusion coefficient. The obtained conversion factor is $c=\left(0.162\pm 0.002\right)\;T{\left(\mathrm{mA}\right)}^{-1}$. This value is in agreement with the value predicted by the gradient coil simulation. It means that pulsed field gradients of up to $16.2\;Tm^{-1}$ may be generated for the NMR diffusion studies with X-nuclei using the maximum available gradient current of 100A. Moreover, the experimentally achieved rise and fall times from $0\;Tm^{-1}$ to maximum gradient strength are 0.16ms and 0.10ms, respectively. These short values are achieved by using exponentially shaped gradient wave forms as described in reference [8]. They are significantly shorter than the nominal values for rise and fall times in commercially available *z*-gradient probes of similar maximum gradient strength and are of advantage when using the system for diffusion studies of fluids with short transverse relaxation times as observed in many porous materials.

 The performance of the modified probe at different resonance frequencies was investigated by PFG NMR measurements of self-diffusion of several monovalent cations (${\mathrm{Li}}^{+}$, ${\mathrm{Na}}^{+}$, ${\mathrm{Cs}}^{+}$) in aqueous solution and of liquid benzene using the NMR active nuclei ${}^{7}\mathrm{Li}$, ${}^{23}\mathrm{Na}$, ${}^{133}\mathrm{Cs}$ and ${}^{13}C$, respectively. For all these different systems, the slope in Figure 2 is found to be linear. Using the current-to-gradient conversion factor obtained by the ${}^{2}H$ PFG NMR measurements, the self-diffusion coefficients were determined. The values are given in Table 1. Within the experimental uncertainty, they agree well with data known for these systems from literature. It shall be noted that—due to the relatively small self-diffusion coefficient of ${\mathrm{Li}}^{+}$ in this aqueous solution and the short observation time of 15ms—it was necessary to apply the maximum pulsed gradient intensity of $16.2\;Tm^{-1}$ to attenuate the ${}^{7}\mathrm{Li}$ NMR spin echo signal amplitude by one order of magnitude as shown in Figure 2. Thus, the positively measured root mean square (r.m.s.) displacements of the ${\mathrm{Li}}^{+}$ cations in this system are 2.6μm.

As for all PFG NMR applications, precise self-diffusion studies with this modified X-observe NMR probe require to obey the filling high of the NMR sample tubes (here 10±1mm) and a correct adjustment of the sample in the isocenter of the gradient coil. The latter was most conveniently done for each sample in these studies prior to the NMR diffusion studies by using a one-dimensional spin echo imaging technique with a read gradient generated with the same gradient coil [10].

#### 2.1.1. Diffusion of ${\mathrm{Li}}^{+}$ and ${\mathrm{Cs}}^{+}$ Cations in Mesocellular Silica Foam

Besides the performance test of the modified X-observe NMR probe, the results for ${\mathrm{Li}}^{+}$ and ${\mathrm{Cs}}^{+}$ self-diffusion in bulk aqueous solution presented in Table 1 serve as reference for comparison with the diffusion behavior of these cations in a mesoporous silica foam (MCF). The successful impregnation of the MCFs by the electrolyte solutions was checked prior to the NMR diffusion studies by visual inspection of the sample texture. Although more than approximately 200-weight% of solution were added to the MCF powder, the individual particles in the bed do not adhere due to capillary forces between outer solid and liquid interfaces. Thus, the presence of significant amounts of external (excess) liquid between the MCF particles can be excluded.

**Table 1:** Diffusion coefficients of monovalent cations in aqueous solutions in bulk and captured in mesocellular silica foam (MCF) as measured by the modified X-observe NMR probe. The data are compared to literature values known for bulk solutions [24]. The ${}^{2}H_{2}O$ data from Weingärtner *et al.* [23] were used to experimentally calibrate the current-to-gradient conversion factor of the modified probe.

Observed X-nucleus and substance	$D\times 10^{9}$ $m^{2}s^{-1}$	*τ*	$D_{\mathrm{ref}}\times 10^{9}$ $m^{2}s^{-1}$	Reference
${}^{7}{\mathrm{Li}}^{+}$ in ${\mathrm{LiCl}}_{ac}$ (bulk)	0.223±0.020	–	0.204	[24]
${}^{7}{\mathrm{Li}}^{+}$ in ${\mathrm{LiCl}}_{ac}$ (MCF)	0.051±0.006	4.4±0.6	–	–
${}^{23}{\mathrm{Na}}^{+}$ in ${\mathrm{NaCl}}_{ac}$ (bulk)	0.95±0.01	–	0.941	[24]
${}^{133}{\mathrm{Cs}}^{+}$ in ${\mathrm{CsCl}}_{ac}$ (bulk)	1.315±0.021	–	1.291	[24]
${}^{133}{\mathrm{Cs}}^{+}$ in ${\mathrm{CsCl}}_{ac}$ (MCF)	0.283±0.045	4.6±0.8	–	–
${}^{2}H_{2}O$ (bulk) – calibration	1.87±0.02	–	1.872	[23]

Due to the high porosity (high specific pore volume of $2.2\;{\mathrm{cm}}^{3}g^{-1}$) of the MCF and the relatively high concentration of the aqueous solutions (about 75% and 85% of the respective saturation concentration at room temperature), the signal-to-noise ratio of the ${}^{7}\mathrm{Li}$ and the ${}^{133}\mathrm{Cs}$ primary spin echo NMR signal is better than 20:1 for a single scan. The total amounts of ${}^{7}\mathrm{Li}$ and ${}^{133}\mathrm{Cs}$ spins in the impregnated MCF samples were $2.41\times 10^{-3}\;\mathrm{mol}$ and $0.95\times 10^{-3}\;\mathrm{mol}$, respectively. By using signal averaging to further improve the signal-to-noise ratio, it is estimated that the modified X-observe probe will be well suited to investigate diffusion of these monovalent cations in aqueous solutions in a concentration range down to about 1/20 of the values used in these experiments. Thus, concentrations of a few percent of the saturation concentration at room temperature are accessible for NMR diffusion studies of these cations in such porous materials.

Due to the good signal-to-noise ratio, spin echo attenuations over more than one order of magnitude were readily measured in the ${}^{7}\mathrm{Li}$ and ${}^{133}\mathrm{Cs}$ PFG NMR experiments with the electrolyte impregnated MCFs. The ${}^{133}\mathrm{Cs}$ NMR data showed a single-exponential decay and were straightforwardly analyzed by using Equation (1). The obtained ${\mathrm{Cs}}^{+}$ self-diffusion coefficient in the MCF is given in Table 1. It is reduced by a factor (*τ*) of about 4.6 compared to the value in the bulk solution. This is due to the restricting influence of the MCF matrix on the Brownian motion of the cations in the solution.

In the corresponding ${}^{7}\mathrm{Li}$ NMR measurements, small contributions from fast bulk cation diffusion were found. Obviously, due to the increased viscosity of the ${\mathrm{LiCl}}_{ac}$ solution, not all of the solution added to the MCF was sucked into the pore space of the silica foam. These contributions were subtracted from the spin echo attenuation before determining the ${\mathrm{Li}}^{+}$ cation diffusion coefficient in the MCF. The result is given in Table 1. With $5.1\times 10^{-11}\;m^{2}s^{-1}$ it is again more than a factor of 4 smaller than the ${\mathrm{Li}}^{+}$ cation diffusion in the bulk aqueous LiCl solution.

It shall be noted that the ${\mathrm{Li}}^{+}$ cation self-diffusion coefficient in the MCF is the smallest self-diffusion coefficient determined with the modified X-observe probe within this paper. The observation time for this measurement was 30ms corresponding to a r.m.s. displacement of $1.7\times 10^{-6}\;m$ in pulsed field gradient direction. This value provides an upper estimate of the displacements, which can positively be measured by the modified X-observe NMR probe.

This displacement is large compared to the pore size but smaller than the particle size of the MCF. Thus, the cations can easily move within the particles. They just experience an averaged obstruction due to the presence of the solid matrix during traveling between the individual pores. If compared to the bulk cation diffusion, the impact of this obstruction is the same for the ${\mathrm{Li}}^{+}$ and the ${\mathrm{Cs}}^{+}$ cations (see Table 1). The ratio of the two diffusivities

(3) $$\tau =\frac{D_{bulk}}{D_{MCF}}=\frac{L^{2}}{L_{0}^{2}}$$

represents the tortuosity *τ* of the pore space. As a geometrical pore structure parameter, *τ* describes the square of the averaged increase of fluid path way through the pore space *L* compared to the direct (straight line) connection $L_{0}$, see second part of Equation 3. A tortuosity of about 4.5 for the MCF is reasonable and means that the cations have to increase their diffusion path through the silica foam by slightly more than a factor of two in order to reach the same displacement as in the bulk solution.

#### 2.1.2. Diffusion of ${\mathrm{CO}}_{2}$ and ${\mathrm{CH}}_{4}$ in MOF CuBTC

In the microporous crystalline metal-organic framework CuBTC, the adsorption capacities for ${\mathrm{CO}}_{2}$ at room temperature and ambient pressure (p≅1bar) are in the range of $4\times 10^{-3}\;{\mathrm{molg}}^{-1}$ [25]. Since the NMR sample tube contains about 150mg of the adsorbent in a bed of 1cm filling height, there are up to about $0.6\times 10^{-3}\;\mathrm{mol}$ ${\mathrm{CO}}_{2}$ in the sensitive NMR volume. Thus, the typical number of NMR active nuclei for the ${}^{13}C$ NMR studies with the adsorbed ${}^{13}C$-enriched ${\mathrm{CO}}_{2}$ is slightly smaller than the number of ${}^{7}\mathrm{Li}$ and ${}^{133}\mathrm{Cs}$ nuclei in the aqueous salt solutions, described above.

**Figure 3:**
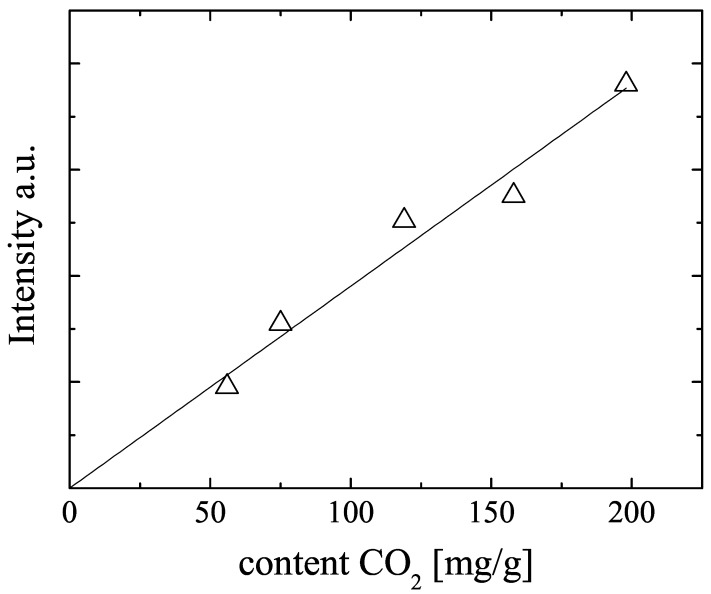
${}^{13}C$ NMR signal intensity observed with a CPMG sequence in dependence on the volumetrically determined amounts of ${\mathrm{CO}}_{2}$ chilled onto the CuBTC mixture samples M1 to M5. The solid line represents the best fit to the data and was used to determine the amount of adsorbed ${\mathrm{CO}}_{2}$ from the ${}^{13}C$ NMR signal intensities (see Table 2).

The modified X-observe probe is capable to observe the ${}^{13}C$ NMR signal of the adsorbed ${}^{13}{\mathrm{CO}}_{2}$ quantitatively. Figure 3 displays the NMR signal intensity, observed with a CPMG sequence [26] of all mixture samples (samples M1–M5 in Table 2) as function of the amount of ${\mathrm{CO}}_{2}$ loaded onto the NMR sample tubes during sample preparation. The plotted signal intensities were obtained by mono-exponential fits of the measured spin echo intensities in the CPMG echo trains. The signal intensities increase monotonously with these ${\mathrm{CO}}_{2}$ amounts, but there is some scatter in the data. Since the NMR signal intensity is proportional to the amount of NMR active nuclei and since the observed NMR signal intensities are found to be reproducibly measured, we recalculated the loading of the samples from the NMR signal intensities. The results are given in Table 2. A similar procedure was applied for the methane loadings which were checked by ${}^{1}H$ NMR using our standard diffusion probe described in [8]. All loadings and the adsorbed ${\mathrm{CO}}_{2}$ molar fraction in Table 2 refer to this corrected values. Deviations from the introduced amounts of the adsorbed gases are caused by incomplete transfer of the volumetrically determined amounts of gases to the NMR sample tubes and scatter in the void volume of the sample tubes above the CuBTC bed, which is filled by gas in equilibrium to the corresponding adsorbed amounts.

**Figure 4:**
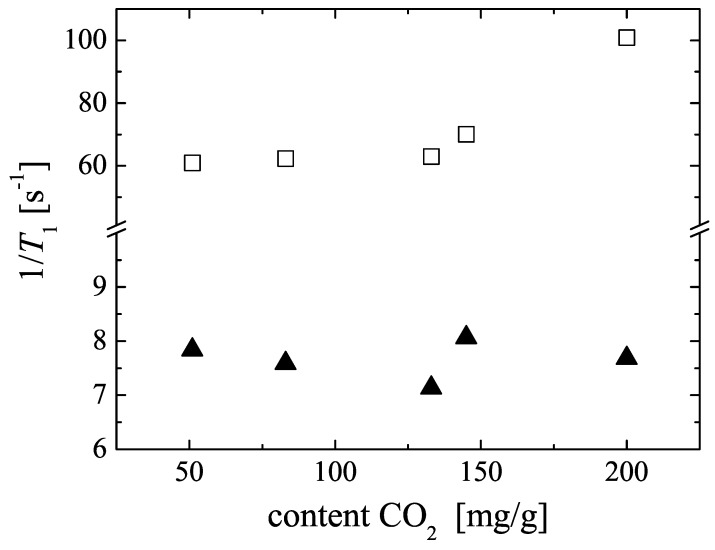
${}^{13}C$ and ${}^{1}H$ longitudinal relaxation rates ($T_{1}^{-1}$) in CuBTC for adsorbed ${}^{13}{\mathrm{CO}}_{2}$ (▲) and ${\mathrm{CH}}_{4}$ (□), respectively. The data are plotted for the mixture samples M1 to M5 in dependence on the amount of adsorbed ${\mathrm{CO}}_{2}$ (see Table 2).

Our own earlier ${}^{1}H$ NMR studies of $C_{3}$ to $C_{6}$ alkanes in MOF CuBTC showed that the longitudinal ($T_{1}$) relaxation times of the adsorbed molecules at room temperature are rather short, which limits NMR diffusion studies to short observation times of 0.8ms≤Δ≤1.4ms using the primary spin echo sequence [27]. Dipole-dipole interactions with the electron spin of framework and extra framework ${\mathrm{Cu}}^{2+}$ in the CuBTC structure and the reduced molecular mobility were identified as reasons for fast relaxation. Figure 4 shows the ${}^{13}C$ and ${}^{1}H$ longitudinal relaxation rates ($T_{1}^{-1}$) of ${}^{13}{\mathrm{CO}}_{2}$ and ${\mathrm{CH}}_{4}$ in the mixture samples. The relaxation rates for ${\mathrm{CO}}_{2}$ are independent of loading and significantly smaller than for ${\mathrm{CH}}_{4}$. The ${}^{13}C$ relaxation times are $T_{1}=\left(130\pm 15\right)\;\mathrm{ms}$ and sufficiently long to apply stimulated spin echo based PFG NMR sequences. The ${}^{1}H$ relaxation rates of methane seem to increase for large carbon dioxide contents, which correspond to large total loadings but small methane contents (see Table 2). We assume that this indicates a reduced mobility of methane due to the presence of a large amount of carbon dioxide in the pore space. The ${}^{1}H$ relaxation times of methane are in the range of 10ms to 18ms, which again limits the applicability of observation time dependent NMR diffusion studies and requires the use of high pulsed field gradient intensities as already known for the longer alkanes [27].

**Figure 5:**
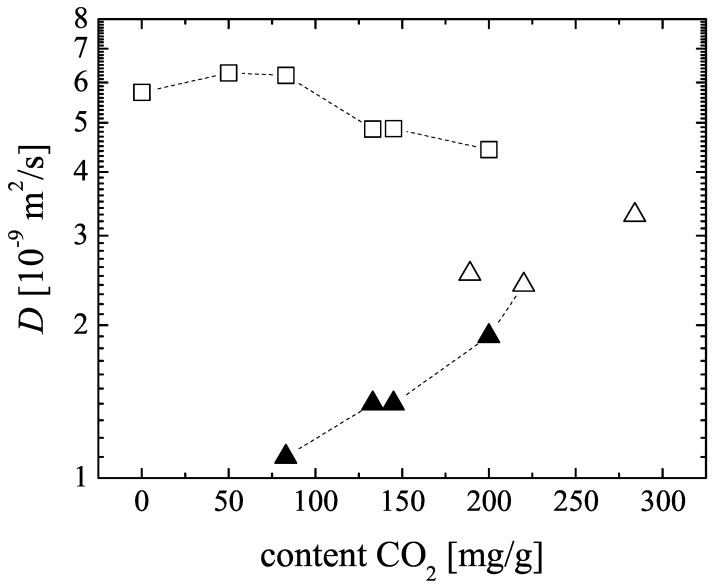
Self-diffusion coefficients *D* in MOF CuBTC in dependence on the adsorbed amount of ${\mathrm{CO}}_{2}$: ${\mathrm{CO}}_{2}$ (▲) and ${\mathrm{CH}}_{4}$ (□) for the co-adsorption of both gases; ${\mathrm{CO}}_{2}$ (△) under single component adsorption. The corresponding methane loadings and total loadings in molecules per u.c. are given in Table 2.

The self-diffusion coefficients of carbon dioxide and methane in CuBTC determined by ${}^{13}C$ and ${}^{1}H$ PFG NMR, respectively, are shown in Figure 5. In all samples, diffusional exchange of the adsorbed phase with the faster diffusing gas phase between the CuBTC crystallites in the bed was observed. The data reported in Figure 5 represent the intracrystalline self-diffusion coefficients, which were obtained from the PFG NMR spin echo attenuations by application of a bi-exponential analog of Equation 1 and assigning the smaller diffusivity to the diffusion of the adsorbed phase.

Single-component ${\mathrm{CO}}_{2}$ diffusivites were measured over a narrow range of loadings and yield self-diffusion coefficients of about $2.5\;\times 10^{-9}\;m^{2}s^{-1}$ with a tendency to increase with loading. According to our knowledge, there are no published data for single-component ${\mathrm{CO}}_{2}$ self-diffusion in CuBTC available. However, results from MD simulations and experimental studies in other MOF systems (IRMOF, MIL, ZIF) show that carbon dioxide is generally highly mobile in these structures and should show an increasing diffusivity with increasing loading, as long as no clustering of ${\mathrm{CO}}_{2}$ molecules in the pore space will occur [4,28,29,30].

For CuBTC, MD simulation predicts for single-component self-diffusion of methane a value of $1.8\;\times \;10^{-8}\;m^{2}s^{-1}$ at room temperature [27]. With $\left(0.58\pm 0.15\right)\times 10^{-8}\;m^{2}s^{-1}$ (see Figure 5 at the zero ${\mathrm{CO}}_{2}$ content) our experimental result is about a factor of three smaller. Possible reasons are a different loading range (higher loading in the experimental study) and a restricting influence of the external crystal surface of the CuBTC MOF on the methane diffusion. Due to the small $T_{1}$ relaxation times of the methane in CuBTC (see Figure 4), this restricting influence could not be evaluated quantitatively by changing the observation time over a sufficiently large interval in the ${}^{1}H$ PFG NMR experiments.

For the experimental ${}^{13}C$ and ${}^{1}H$ PFG NMR diffusion with co-adsorbed carbon dioxide and methane the samples were prepared under such conditions that the total pressure in the gas phase above the CuBTC bed is not exceeding but close to 1bar. In Figure 5, the corresponding data points including the single-component results for zero and $220\;\mathrm{mg}\;g^{-1}$ ${\mathrm{CO}}_{2}$ content are connected with a dashed line. With increasing ${\mathrm{CO}}_{2}$ content, the diffusivity of carbon dioxide in the mixture increases resuming the trend observed for the single-component ${\mathrm{CO}}_{2}$ data. The self-diffusion coefficients for the co-adsorbed methane, which always diffuses faster than the ${\mathrm{CO}}_{2}$ in the considered range of loadings, are found to decreases slightly.

From experimental studies and computer simulation it is known that carbon dioxide is more strongly adsorbed in CuBTC than methane [13,25,31,32]. At small carbon dioxide contents, most of the carbon dioxide is attracted to strong adsorption sites. The co-adsorbed methane cannot compete in host-guest interaction. Thus, methane diffusion in the potential of the MOF framework is faster than carbon dioxide diffusion. With increasing carbon dioxide loading, the averaged interaction with the host lattice becomes smaller since the strongest adsorption sites are already covered and the carbon dioxide diffusion increases. This behavior is in agreement with such pattern of loading dependent diffusivities in microporous materials, where increasing amounts of molecules strongly interacting with the host system reduce the potential barrier on the diffusion path through the pore space or the life time in the transition state [3].

With increasing carbon dioxide content (which corresponds to a decreasing methane content), the diffusion path of methane in the pore space is influenced by the presence of more and more molecules occupying the pore space. The majority of these molecules is slowly diffusing ${\mathrm{CO}}_{2}$. Thus the free volume for methane is continuously reduced, leading to the observed slightly decreasing methane diffusion coefficients.

The ratio between the methane and the carbon dioxide diffusivities, which is called the diffusion separation factor ($D_{{\mathrm{CH}}_{4}}/D_{{\mathrm{CO}}_{2}}$), is important for estimating the performance of microporous materials in gas separation processes. Figure 5 and Table 2 show that this ratio depends mainly on the adsorbed carbon dioxide content. It decreases from about 5.6 at an adsorbed carbon dioxide molar fraction of $\chi_{{\mathrm{CO}}_{2}}=0.77$ to 2.3 at $\chi_{{\mathrm{CO}}_{2}}=0.97$. Using only the experimental single-component self-diffusion coefficients one obtains also a value of about 2.3.

Several computer simulations of the performance of CuBTC in ${\mathrm{CH}}_{4}/{\mathrm{CO}}_{2}$ gas separation applications report on separation factors [22,33]. For methane and carbon dioxide, Keskin *et al.* [34] investigated also diffusion separation factors, without reporting the corresponding single-component self-diffusion coefficients in the mixture. In this mixture MD simulation study, where the molar ratio of both gases in the gas phase is 1:1, a cross-over from $D_{{\mathrm{CH}}_{4}}/D_{{\mathrm{CO}}_{2}}=2.3$ at pressures p≤2bar to smaller than unity is observed at a total pressure of about 8bar [34] (corresponding to 4bar ${\mathrm{CO}}_{2}$ partial pressure). The cross-over in the ratios of the self-diffusion coefficients means that the ${\mathrm{CO}}_{2}$ is expected to diffuse faster at high total loadings than the ${\mathrm{CH}}_{4}$.

Our experimental results for $D_{{\mathrm{CH}}_{4}}/D_{{\mathrm{CO}}_{2}}$ at p≤1bar agree with these MD predictions (see Table 2). Extrapolating our NMR data in Figure 5 towards higher carbon dioxide contents, we expect the cross-over at a carbon dioxide loading of about $300\;\mathrm{mg}\;g^{-1}$. Using the adsorption isotherm published by Martin-Calvo *et al.* [33], the corresponding partial ${\mathrm{CO}}_{2}$ pressure in the gas phase is estimated to be about3–4bar. Thus, our observed dependence of the self-diffusion coefficients on adsorbed ${\mathrm{CO}}_{2}$ content confirms the cross-over of the diffusion separation factor predicted by the mixture MD simulations as well. It will be an interesting challenge for future experimental PFG NMR studies with the modified X-observe probe to explore directly the diffusion of ${\mathrm{CO}}_{2}$ containing gas mixtures in MOFs and other microporous materials in the high-pressure range.

## 3. Experimental Section

### 3.1. Gradient Coil Design for X-Observe NMR Probe

The Maxwell-type *z*-gradient coil for the WB 400 X-observe NMR probe (Bruker, Germany) was designed to match the requirements and restrictions mentioned in Section 2.1. To optimize the coil design, a finite element simulation of the magnetic flux density for a proposed current distribution of the coil was performed using the commercial software COMSOL Multiphysics^®^ (COMSOL AB, Sweden). The axial symmetric model was used and time-independently solved for the vector potential of the azimuthal induction currents. For calculations, only the positions of the windings of the copper wire (0.45mm diameter) carrying the current were taken into account. Coil support materials and surrounding structures were treated as materials having the magnetic permeability of air. The solution was displayed for the gradient of the *z*-component of the magnetic flux density $g_{z}\left(r,z\right)$ and the magnitude of the magnetic flux density B(r,z) in surface plots and contour plots, respectively as shown in Figure 1 b.

During the design process, the solutions of the simulations were evaluated. By varying the positions, the numbers and distribution of the copper windings of the coil, the strength and homogeneity of the magnetic field gradient was successively improved. It turned out, for example, that the separation of a few windings from each of the two main blocs towards the center of the coil in *z*-direction improves the homogeneity of the field gradient significantly.

The ohmic resistance *R* of the coil was monitored to keep it below 2Ω. This was necessary in order to be able to use the full power of our gradient current amplifier [8]. Also, the magnitude of the magnetic flux density B(r,z) at the position of the metal probe cover at the radius of r=35mm was observed to check for possible generation of eddy currents in the metal structure during pulsed field gradient operation.

The result of the design process, shown in Figure 1 b, is a coil with 2×52 windings at a mean radius of r=14mm, separated in one large block with 3 layers and one smaller block with 2 layers of windings. This gradient coil is wound on the coil support which is made of polyether ether ketone (PEEK). The inner diameter of the coil support is 22mm, which is well suited to accommodate the X-band RF coil of the WB 400 NMR probe and a Teflon tube for thermal insulation of the gradient coil from the active sample volume in the center. The coil was manufactured and wound in the workshop of our institute. The achieved values for its ohmic resistance and inductance are R=1.13Ω and L=160μH, respectively. As the experimentally determined current-to-gradient conversion factor of $c=\left(0.162\pm 0.002\right)\;T{\left(m\;A\right)}^{-1}$ (see Section 2.1), also these values agree with the results predicted from the coil simulation.

In order to accommodate and fix the gradient coil in the WB 400 probe, material was removed by turning from its upper and lower ceramic support parts. The standard glass dewar system of the WB 400 was removed and replaced by the above described Teflon tube for thermal insulation. An air cooling system was added to dissipates the Joule heat generated during pulsed gradient operation. These modifications reduce the temperature variability of the modified X-observe probe to 293≤T/K≤333 with an accuracy of ±1.5K. To connect the gradient coil to the gradient current amplifier, current leads were added. In order to reduce RF noise received via these current leads, which would disturb the observed NMR signal, the probe internal leads were equipped with small capacitors connected to ground, shortcutting them for received RF. The major modifications of the X-observe NMR probe are indicated in the photograph in Figure 1 a.

### 3.2. Salt Solutions and Bulk Liquids as Reference Materials

Aqueous solutions of LiCl, NaCl and CsCl were prepared with concentrations of $14.9\;\mathrm{mol}\;L^{-1}$, $4.3\;\mathrm{mol}\;L^{-1}$ and $9.5\;\mathrm{mol}\;L^{-1}$, respectively. These concentrations are less then 90% of the saturation concentration of the salts in water at 273K. They were chosen since reference data for cation diffusion are available in literature [24]. Additionally, these concentrations ensure that the salts will neither precipitate at the NMR measurement temperature of (298.3±1.5)K nor during long storage periods at room temperature. Isotope labeled deuterated water (99.95%) and ${}^{13}C$-enriched benzene (35% enrichment) were purchased from Chemotrade (Germany) and used as received. All liquids were introduced into NMR sample tubes, obeying a filling hight of about 1cm. The tubes were flame sealed to prevent fluid loss by evaporation.

### 3.3. Mesocellular Silica Foams for NMR Studies

**Figure 6:**
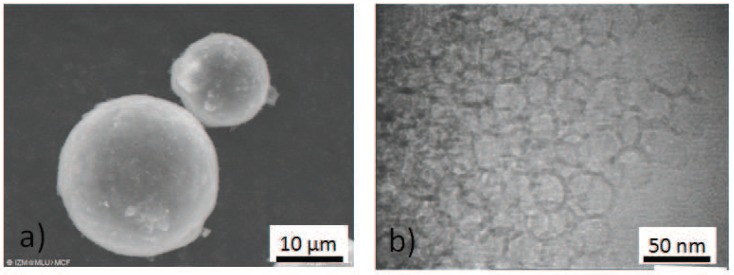
SEM (**a**) and TEM (**b**) of the mesocellular foams investigated by PFG NMR diffusion studies. The MCF consists of spherical particles with almost spherical pores. The pore walls are thin leading to a high specific pore volume.

Mesocellular silica foams are pure siliceous materials with a very high specific pore volume [14,18]. They are synthesized from TEOS as silica source under slightly acidic, hydrothermal conditions. Pluronic-123 and Mesitylene, which are the structure forming agents in the synthesis solution, are completely removed after synthesis by calcination. The specific pore volume of our MCF was $2.2\;{\mathrm{cm}}^{3}g^{-1}$. According to SEM and TEM analysis (see Figure 6), the material consists of almost spherical particles of 5 to 10μm particle size containing pores of about 2nm diameter. These pores are well interconnected by holes of up to 0.7nm diameter. Due to the large pore size and the relatively fragile pore wall, nitrogen adsorption and mercury intrusion porosimetry are only to a limited extent applicable for pore structure characterization.

Therefore, PFG NMR was applied to probe the connectivity between the pores and the homogeneity of the MCF material. The calcinated, dry MCF was introduced into NMR sample tubes up to a filling hight of 1cm. This corresponds to about 30mg of the foam. The tubes were evacuated and volumetrically determined amounts of aqueous solutions of LiCl and CsCl (concentrations see above) were added to the MCF bed. The NMR tubes were flame sealed immediately, centrifuged and stored at 353K for 24h. After this procedure, the bed of the impregnated MCF in the NMR tubes appeared dry, which means that the aqueous salt solutions were successfully sucked into the pore space.

### 3.4. Preparation of CuBTC Samples

In CuBTC (Cu${}_{3}$(BTC)${}_{2}$, BTC = 1,3,5-benzentricarboxylate) [20], Cu${}^{2+}$ dimers form the metal centers which are surrounded and three-dimensionally interconnected by the BTC linkers. According to Getzschmann *et al.* [35], the microporosity of CuBTC is formed by three types of pores, two large pores with diameters of 10.5 and 12.4 Å and one small pore (side pocket) with a diameter of 4.9 Å. This microporous metal-organic framework is commercially available from Sigma-Aldrich as Basolite^®^ C 300 (produced by BASF). Basolite^®^ C 300 has an average crystal size of 16μm and a BET surface area of 1500–2100 $m^{2}/g$ [20,36].

**Table 2:** Single-component (S) and mixture (M) adsorption samples prepared for NMR studies on CuBTC: CO${}_{2}$ and CH${}_{4}$ loadings, molar fraction of adsorbed CO${}_{2}$ ($\chi_{{\mathrm{CO}}_{2}}$), total number of molecules per unit cell (u.c.) and ratio of NMR measured self-diffusion coefficients ($D_{{\mathrm{CH}}_{4}}/D_{{\mathrm{CO}}_{2}}$).

Sample ID	CO${}_{2}$	CH${}_{4}$	$\chi_{{\mathrm{CO}}_{2}}$	Molecules	$D_{{\mathrm{CH}}_{4}}/D_{{\mathrm{CO}}_{2}}$
	$\mathrm{mg}\;\cdot \;g^{-1}$	$\mathrm{mg}\;\cdot \;g^{-1}$		total / u.c.	
S1	189	–	–	35	–
S2	220	–	–	42	–
S3	284	–	–	53	–
S4	–	15	–	8	–
M1	50	12.5	0.59	16	–
M2	83	9.1	0.77	20	5.6
M3	133	8.6	0.85	29	3.5
M4	145	5.3	0.91	30	3.5
M5	200	2.2	0.97	39	2.3

NMR samples were prepared from Basolite^®^ C 300. For each sample an amount of 130–190 mg of CuBTC was filled into an NMR sample tube. The samples were slowly heated in vacuum up to a temperature of 403K, which was maintained for 24h to remove residual solvents, gases and moisture from the pore space. After controlled cooling down to room temperature, the single-component adsorption (samples S1–S4, Table 2) was performed by exposing the activated CuBTC to volumetrically determined amounts of ${\mathrm{CO}}_{2}$ (99% ${}^{13}C$-enriched, Sigma-Aldrich) and ${\mathrm{CH}}_{4}$, respectively. The required amounts of the adsorbate gases were determined using published single-component adsorption isoterms [25]. The gases were frozen into the sample tube by chilling the CuBTC bed to about 77K using liquid nitrogen. Finally, the glass tubes were sealed by ablating them a few centimeter above the CuBTC bed.

The five samples containing a mixture of ${\mathrm{CO}}_{2}$ and ${\mathrm{CH}}_{4}$ (samples M1–M5, Table 2) were prepared in a similar way using two successive adsorption steps. First, the carbon dioxide was adsorbed as described above. The samples were kept at 77K until in the second step, the corresponding volumetrically determined amount of methane was co-adsorbed. The achieve adsorbed amounts were checked by the observed NMR signal intensities and corrected in such a way that they are proportional to the observed NMR signal intensities. The results of this correction represent the actually achieved adsorbed amounts and are reported in Table 2.

### 3.5. NMR Diffusion and Relaxation Measurements

All X-nuclei NMR studies were performed on the home-built spectrometer *FEGRIS FT* operating at a magnetic flux density of $B_{0}=9.4\;T$ using the modified X-observe probe described above. The corresponding ${}^{1}H$ NMR studies with methane adsorbed in CuBTC were performed on the spectrometer *FEGRIS NT* operating at a magnetic flux density of $B_{0}=2.9\;T$ with the NMR probe system described in reference [8]. For the NMR diffusion studies of the bulk liquids and of the monovalent cations in aqueous salt solutions, the primary spin echo sequence with one pair of pulsed field gradients was applied [6,7]. The PFG NMR diffusion studies in the porous systems were performed using the 13-interval pulse sequence [8,37]. The 90°–180° rf pulse distance was set to 2ms in the measurements of the cation diffusion in MCF and to 1.2ms in the measurements with the CuBTC. Under these conditions, typical values for the width of the pulsed field gradients are about 0.5ms to 0.8ms. The diffusion times were generally varied between 10ms and about 30ms. These values are much shorter than the longitudinal relaxation times of the systems so that relaxation time weighting of the observed NMR signals is negligible. For the CuBTC studies, NMR signal intensity measurements and $T_{1}$ relaxation time measurements were carried out using the CPMG [26] and Inversion recovery sequences, respectively.
